# Fevers

**Published:** 1905-12-23

**Authors:** 


### FEVERS.

(*Continued from page* 185.)

**Some Tropical Fevers.**—The physical evils entailed by a great war are incontestable, yet terrible as is the picture of carnage and death from disease and vast as is the tale of resulting ill-health, some good to medicine, and consequently to humanity, may result. The study of a disease is stimulated by the contemplation of its ravages on a large scale, and the investigation of new complaints is initiated by contact with them. The effect on medicine in America by the acquisition of tropical territory by the United States as a result of the

Spanish-American War formed the subject of an address by G. Blumer [11] of San Francisco at the last annual meeting of the American Medical Association. The increased military and commercial intercourse thus resulting from the acquisition of Porto Rico and the Philippine Islands has its dangers as well as its advantages. In a wide area like that of the States, which includes sub-tropical regions, the introduction by returned soldiers and others of hitherto unknown diseases from the tropics is to be feared and guarded against. On the other hand facilities are greatly increased for the study of these diseases and their investigation may lead to the detection of hitherto unsuspected cases in the home country. Thus, to take one example, prior to 1900 only ten authentic cases of *uncinariasis* or ankylostomiasis had been reported in the States, while since this date there have been recorded over 1,200 cases in which the hook-worm was found scattered over a wide area. The detection of these sporadic cases is, of course, of great importance, and Blumer thinks the mental and physical deterioration resulting from this infection explains the character for laziness and slackness earned by the "poor whites" of the south. The knowledge of *dysentery* has been greatly advanced by the American occupation. The division into two groups, that of amœbic dysentery with which is associated tropical abscess, and that due to the bacillus of Shiga or of Flexner is well known. The later discovery that an organism similar to that producing tropical bacillary dysentery is found in the non-tropical disease and in certain of the summer diarrhœas of children is of much interest. In infantile diarrhœa associated with mucus and blood in the stools the bacillus dysenteriæ is commonly found, and acting on Flexner's suggestion, attempts have been made to produce a curative serum. Results so far have been disappointing, but it is hoped that in time a serum will be found, the early use of which will be effectual in combating this scourge of town-bred babies. The brilliant results attending the establishment of Finlay's contention that *yellow fever* infection is carried by a species of mosquito, and the wonderful effect following the practical expression of this belief in Havana are now matters of history. But we are still ignorant of the cause of the disease, and vigilance and care are greatly needed. A closer study of *malaria* in soldiers returned from the Philippines overthrew in the minds of American physicians the existence of typho-malarial fever, and showed that typhoid fever and malaria were rarely co-existent. In the majority of infections with malaria, often latent or masked, the æstivo-autumnal crescent was found by Craig. Members of the Yellow Fever Institute have shown the presence of a toxin in the blood at the time of the chill and have produced a chill by the intravenous injection of filtered blood taken at the height of a paroxysm. Ronald Ross [12] has shown by a reduction of the question to stern mathematics and the law of probabilities that, notwithstanding immigration of mosquitoes into the cleared area, good results certainly follow from the destruction of the insects, and especially of their larvæ, in definite restricted areas, even in infected zones. The larger the cleared area the less will it be encroached on, naturally, by immigrant insects. The territories acquired from Spain seem to be a happy hunting ground for most of the tropical diseases, for malta fever, dengue, asiatic cholera, trypanosomiasis, chiefly in the form of kala-azar, and filariasis have all furnished opportunities for study there. The epidemic of cholera in 1902 caused over 90,000 deaths, chiefly among the native populations, in Manilla and the neighbouring island before it was stamped out. R. P. Strong succeeded in producing a protective serum less irritating than that of Haffkine, but it was obtained too late for extended use in this epidemic. From another part of the world, the Federated Malay States, some interesting notes on seven fatal cases of *beri-beri* are furnished by P. N. Gerrard.[13] All the fatalities occurred in the same hospital ward, in patients previously under treatment for less acute beri-beri, with the exception of one man brought in moribund, and, with one exception, no other patient in the ward or hospital not previously admitted for the disease developed it. All the patients were being dieted in the same way as the 400 other inmates of the hospital. The seven cases were all adult males ranging in age from twenty-three to fifty-three. Fulminating symptoms ended in death usually in a few hours; in one case the duration was four days. The onset of the crisis was in various cases marked by rise of temperature, increased abdominal reflexes, splenic and general abdominal tenderness, diminished passage of urine, and congestion of the mucous membranes in addition to the more classical signs, such as paralytic over-distension of the heart, and epigastric pain and fulness. Several showed signs of pericarditis or pericardial effusion. Post-mortem cardiac dilatation, sometimes enormous, and more or less general signs of engorgement and congestion of the various viscera were found. Of less well-known symptoms may be mentioned marked congestion and erosion of the gastric mucosa, usually best marked at the pyloric end, extending into the duodenum and in one instance appearing as inflammatory patches at intervals throughout the small and large bowel as far as the anus. In treatment the exhibition of alkalies, pushed in one case apparently with the idea of neutralising any acid toxin which might be present in the alimentary canal or blood, appeared harmful and was followed by diminished excretion of urine and increased œdema. This result recalls recent teaching on the reduction of dropsical effusions which is said to take place on the elimination of sodium chloride from the diet. Bleeding appeared of no permanent value, and saline infusions, given with the hope of diluting the poison in the blood, were ineffectual. The exhibition of the latter, however, could hardly be expected to be beneficial once the mechanical conditions of an over-distended right heart were set up. A case of *blackwater fever*, under the care of E. S. Crispin,[14] may be mentioned. It occurred during an ordinary attack in a malarial subject accustomed to take as much as sixty grains of quinine a day. The treatment adopted was hypodermic injections of quinine. The hæmoglobinuria ceased within twenty-four hours of the last injection, and the patient died three days later from weakness and return of fever. No parasites and very little malarial pigment were found post-mortem in the spleen, which was almost fluid. Yellow pigment but no true malarial pigment was present in the liver which was shrunken, hard, and showed microscopically atrophy, cloudy swelling and degeneration of the cells, changes recalling some of those found in acute yellow atrophy of the liver.

[11] Med. Rec., July 22. [12] Brit. Med. Jour., May 13. [13] Lancet, June 17. [14] Ibid., August 5. [15] Ibid.

